# Minocycline treatment increases resistance to oxidative stress and extends lifespan in *Drosophila* via FOXO

**DOI:** 10.18632/oncotarget.21224

**Published:** 2017-09-23

**Authors:** Gang Jun Lee, Jin Ju Lim, Seogang Hyun

**Affiliations:** ^1^ Department of Life Science, Chung-Ang University, Seoul, Korea

**Keywords:** minocycline, anti-aging, Foxo, Drosophila

## Abstract

Minocycline is a semi-synthetic tetracycline derivative antibiotic that has received increasing attention for its non-antibiotic properties, mainly anti-inflammatory, tumor-suppressive, and neuroprotective effects. *Drosophila* is a widely used genetically tractable model organism for studying organismal aging by virtue of its short lifespan and ease of cultivation. In this study, we examined the effects of minocycline on *Drosophila* lifespan and its associated traits. Minocycline-supplemented food significantly extended lifespan in both *Canton S* and *w^1118^ Drosophila* strains. The drug-induced lifespan extension was not associated with reduced dietary intake or reduced female fecundity, but rather with increased resistance to an oxidative stressor (hydrogen peroxide). Notably, minocycline's effects on lifespan and resistance to oxidative stress were largely abrogated in *Forkhead box O* (*FOXO*) null mutant, and the drug treatment increased the activity of FOXO. These results may further our understanding of minocycline's beneficial effects against several age-associated deteriorations observed in animal models.

## INTRODUCTION

Minocycline (7-dimethylamino-6-desoxytertracycline) is a second-generation, semi-synthetic tetracycline analog that has been used for over 30 years to treat various symptoms related to bacterial infections, including acne vulgaris and some sexually transmitted diseases [1, 2]. Minocycline, capable of being easily absorbed into the body and displaying a long half-life and excellent tissue penetration, shows a better pharmacokinetic profile than some tetracyclines when used orally [3]. Moreover, minocycline is a highly lipophilic molecule that can easily pass through the blood-brain barrier [2, 4, 5].

Recent work has focused on the non-antibiotic properties of minocycline, such as its anti-inflammatory and anti-apoptotic activities, as well as inhibition of proteolysis and suppression of cancer metastasis [6–9]. These features support the potential use of the drug in the treatment of rheumatoid arthritis, inflammatory bowel syndromes, scleroderma, aortic aneurysms, and malignant tumors [6, 10, 11]. Furthermore, due to its ability to cross the blood-brain barrier to reach the central nervous system, minocycline has been shown to be an effective therapeutic option in various experimental models of brain injury, as well as in several neurodegenerative conditions such as Parkinson's disease, Huntington's disease, amyotrophic lateral sclerosis, and Alzheimer's disease [12–17]. Although the mechanism of action underlying the antibiotic properties of minocycline is largely related to its ability to bind to the bacterial 30S ribosomal subunit, thereby inhibiting protein synthesis, the exact mechanisms of action underlying its non-antibiotic properties (anti-inflammatory, immunomodulatory, and neuroprotective effects), are not yet understood.

Aging can largely be regarded as the progressive decline in physiological integrity and function, which includes molecular interactions, cellular functions, tissue structure and function, and systemic physiological homeostasis [18]. This age-associated decline is accompanied by a progressive increase in mortality and a spectrum of age-related conditions such as diabetes, cardiovascular diseases, and neurodegenerative disorders. However, genetic studies from several model organisms have established that aging is a plastic process, such that manipulation of even a single gene among several conserved genetic pathways, such as insulin/TOR signaling, can dramatically modify the rate of aging and extend lifespan [19]. *Forkhead box O* (*FOXO*) is the transcription factor acting as downstream effector of insulin signaling; FOXO activity has been shown to be associated with stress resistance and lifespan extension in many organisms including human [19].

*Drosophila melanogaster* has been widely used in aging research for its relatively short lifespan (approximately 3 months), low maintenance requirements, and large number of available experimental tools that enable *in vivo* genetic manipulation. Moreover, more than 75% of known human disease genes, covering a broad range of disorders, have fly homologs [20]. These characteristics highlight *Drosophila* as an ideal model organism for investigating the mechanisms of aging and for developing therapeutic interventions that treat human aging and its associated pathologies.

In this study, we tested the potential effects of minocycline on the aging process in *Drosophila*. Feeding minocycline to adult *Drosophila* significantly extended lifespan, which was consistently seen in both *Canton S* and *w^1118^ Drosophila* strains regardless of sex. Minocycline-induced extension of lifespan in the *w^1118^* strain, which is defective in intracellular transport of tryptophan [21], does not support a previously suggested hypothesis that the longevity effect of the drug was conferred via the kynurenine pathway [22, 23]. We found that minocycline treatment neither decreased the rate of food consumption nor impaired the fecundity of flies. However, the flies fed minocycline exhibited increased resistance to hydrogen peroxide treatment, associating a long lifespan with oxidative stress resistance. Notably, the drug's effects on lifespan and oxidative stress resistance were largely absent by mutation of *FOXO*, a key gene in the aging and stress resistance pathways; minocycline treatment generally increased the activity of FOXO, providing insights of minocycline's underlying mechanism.

## RESULTS

### Minocycline feeding extends lifespan in both *Canton S* and *w^1118^ Drosophila* strains

**Figure 1:**
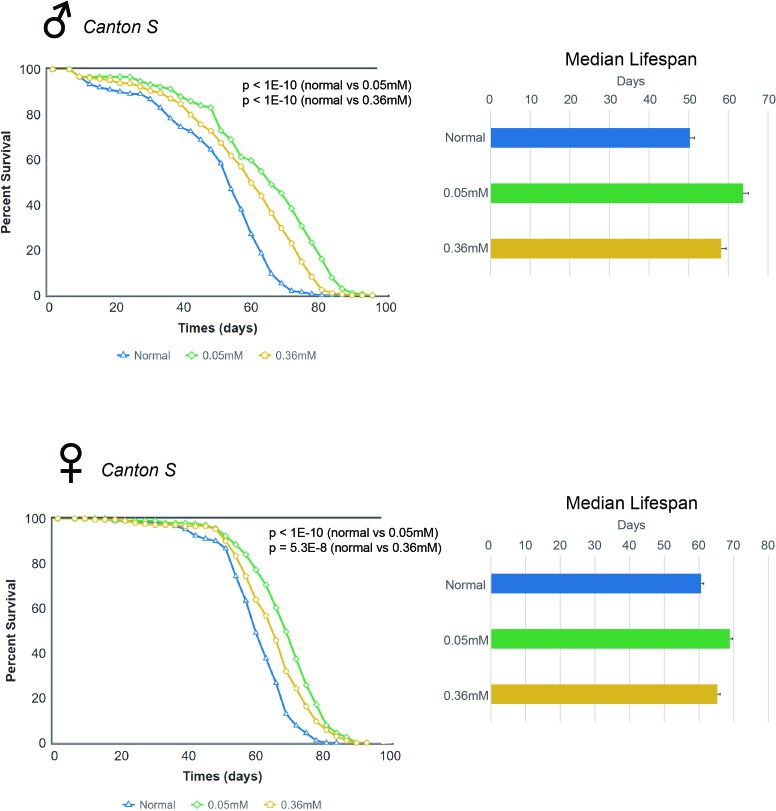
Minocycline extends lifespan in *Canton S Drosophila* strain Minocycline treatment (0.05 mM and 0.36 mM) extends lifespan of *Canton S* flies, as shown by the survival curve (*left*) and by quantification of median lifespan (*right*). A more pronounced lifespan extension was induced by the low dose (0.05 mM). The longevity effect of the drug was seen in both male (*upper panel*) and female (*lower panel*) flies. For male flies, n = 224 (normal), n = 222 (0.05 mM), n = 225 (0.36 mM); *p* < 1.0 × 10^−10^ (both normal vs. 0.05 mM and normal vs. 0.36 mM). For female flies, n = 226 (normal), n = 223 (0.05 mM), n = 220 (0.36 mM); *p* < 1.0 × 10^−10^ (normal vs. 0.05 mM), *p* = 5.3 × 10^−8^ (normal vs. 0.36 mM). *p*-values for lifespan curves were calculated by the log-rank test. All lifespan experiments were repeated with similar results; representative experiments are shown. Data for median lifespan are presented as mean ± SEM.

We examined the effect of minocycline on the lifespan of adult *Drosophila* from two commonly used strains, *Canton S* and *w^1118^*. Previous studies noted the effect of minocycline treatment on the *Oregon R* strain of *Drosophila*, showing that the drug can extend lifespan in this strain [22, 24]. We found that minocycline at either a low dose (0.05 mM) or high dose (0.36 mM) significantly increased lifespan in *Canton S* flies, and this effect was seen consistently in both male and female flies (Figure 1 and Supplementary Figure 1). Male *Canton S* flies fed normal food had a median lifespan of 50.29 d, which increased by 26.7% (63.71 d) or by 15.7% (58.18 d) with a low dose (0.05 mM) or high dose (0.36 mM) of minocycline, respectively. Female *Canton S* flies fed normal food had a median lifespan of 60.6 d, which increased by 13.6% (68.87 d) or by 7.7% (65.26 d) with a low dose (0.05 mM) or high dose (0.36 mM) of minocycline, respectively. In both male and female flies, a low dose of minocycline resulted in more pronounced effects of lifespan extension than the high dose, suggesting that lifespan-extending effects of minocycline in *Drosophila* can be maximized by determining the optimum dose. Moreover, although lifespan was extended in both male and female flies by minocycline treatment, male flies exhibited more robust extension of lifespan than female flies.

**Figure 2:**
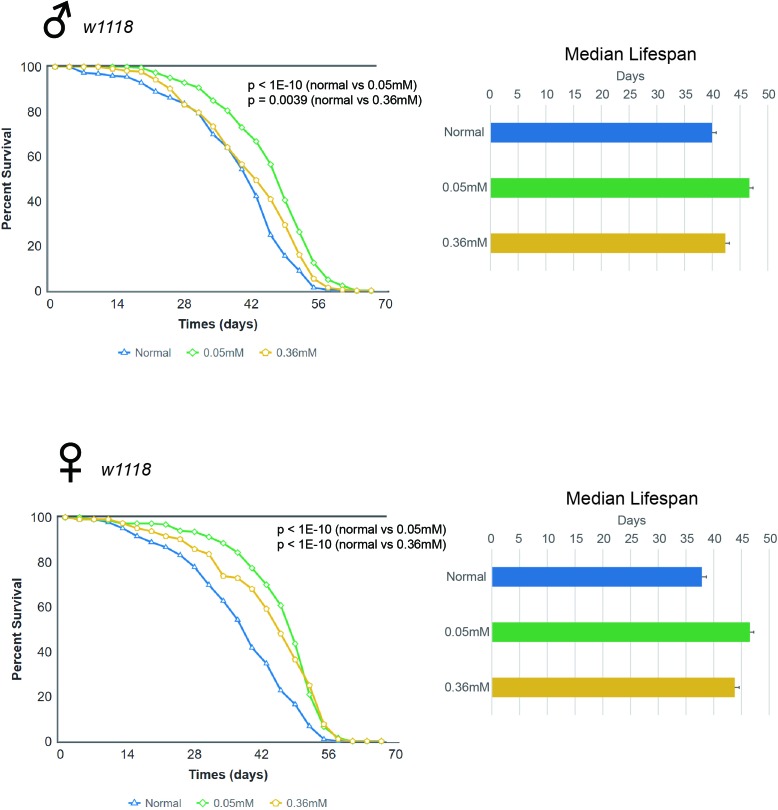
Minocycline extends lifespan in *w^1118^ Drosophila* strain Minocycline treatment (0.05 mM and 0.36 mM) extends lifespan of *w^1118^* flies, as shown by the survival curve (*left*) and by quantification of median lifespan (*right*). A more pronounced lifespan extension was induced by the low dose. The longevity effect of the drug was seen in both male (*upper panel*) and female (*lower panel*) flies. For male flies, n = 228 (normal), n = 230 (0.05 mM), n = 220 (0.36 mM); *p* < 1.0 × 10^−10^ (normal vs. 0.05 mM), *p* = 0.0039 (normal vs. 0.36 mM). For female flies, n = 225 (normal), n = 232 (0.05 mM), n = 220 (0.36 mM); *p* < 1.0 × 10^−10^ (both normal vs. 0.05 mM and normal vs. 0.36 mM). *p*-values for lifespan curves were calculated by the log-rank test. All lifespan experiments were repeated with similar results; representative experiments are shown. Data for median lifespan are presented as mean ± SEM.

Next, we examined the longevity effects of minocycline in the *w^1118^ Drosophila* strain. Similar to findings in *Canton S* flies, we found that the lifespan of *w^1118^* flies fed minocycline at either the low or high dose increased significantly, and this effect was seen in both male and female flies (Figure 2 and Supplementary Figure 2). Male *w^1118^* flies fed the control food had a median lifespan of 39.95 d, which increased by 16.9% (46.69 d) or by 6.0% (42.36 d) with a low or high dose of minocycline, respectively. Female *w^1118^* flies fed the control food had a median lifespan of 37.87 d, which increased by 22.9% (46.53 d) or by 15.6% (43.79 d) when treated with a low or high dose of minocycline, respectively. As was the case with *Canton S* flies, *w^1118^* flies also exhibited a more robust lifespan extension with a low dose of minocycline (0.05 mM). Interestingly, however, the more pronounced minocycline effect on lifespan in males seen in *Canton S* flies was not seen in *w^1118^* flies. Furthermore, minocycline treatment appeared to more strongly increase median lifespan than maximum lifespan in female *w^1118^* flies, which contrasts with the similar effects of the drug on median and maximum lifespan seen in male *w^1118^* and *Canton S* flies.

Since previous studies showed that minocycline treatment increased locomotive activity in *Oregon R* male flies, we tested whether similar effects of the drug were also seen in *Canton S* and *w^1118^* flies. We found that minocycline stimulated the locomotive activity more strongly in male flies than in female flies and in *Canton S* flies than *w^1118^* flies; minocycline failed to increase locomotive activity in *w^1118^* female flies (Supplementary Figure 3).

Taken together, these results suggest that minocycline treatment can extend lifespan and improve motor activity in *Drosophila* strains with a broad spectrum of genetic backgrounds. The observations in the *w^1118^* strain also indicate that the drug can exert longevity effects independently of the kynurenine pathway (see below).

### Minocycline feeding extends lifespan in germ-free *w^1118^ Drosophila* strains

**Figure 3:**
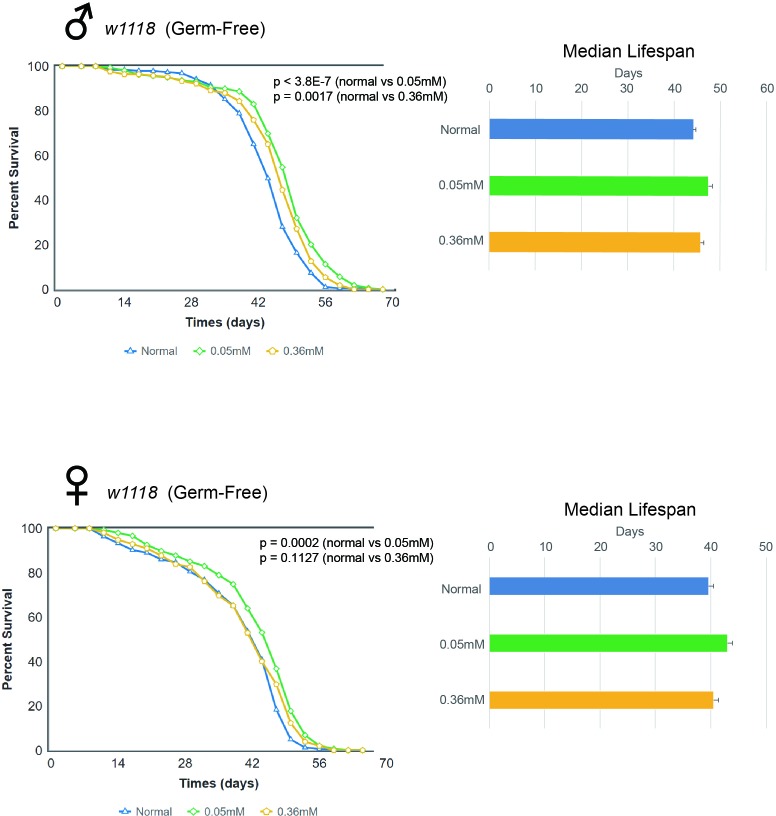
Minocycline extends lifespan in germ-free *w^1118^ Drosophila* strain Minocycline treatment (0.05 mM and 0.36 mM) extends lifespan of germ-free *w^1118^* flies, as shown by the survival curve (*left*) and by quantification of median lifespan (*right*). A more pronounced lifespan extension was induced by the low dose. The longevity effect of the drug was seen in both male (*upper panel*) and female (*lower panel*) flies. For male flies, n = 100 (normal), n = 100 (0.05 mM), n = 100 (0.36 mM); p < 3.8 × 10^−7^ (normal vs. 0.05 mM), p = 0.0017 (normal vs. 0.36 mM). For female flies, n = 100 (normal), n = 100 (0.05 mM), n = 100 (0.36 mM); p = 0.0002 (normal vs. 0.05 mM), p = 0.1127 (normal vs. 0.36 mM). p-values for lifespan curves were calculated by the log-rank test. All lifespan experiments were repeated with similar results; representative experiments are shown. Data for median lifespan are presented as mean ± SEM.

We then investigated whether the lifespan extending effects of minocycline was due to its antibiotic effects. To do this, we established the germ-free rearing condition of *Drosophila* and performed similar experiments of monitoring lifespan as above. We found that the lifespan of germ free *w^1118^* flies fed minocycline at either the low or high dose still increased significantly and this effect was seen in both male and female flies (Figure 3). Male germ-free *w^1118^* flies fed the control food had a median lifespan of 44.05 d, which increased by 7.8% (47.52 d) or by 4.4% (46.02 d) with a low or high dose of minocycline, respectively. Female germ-free *w^1118^* flies fed the control food had a median lifespan of 39.05 d, which increased by 7.8% (42.10 d) or by 4.2% (40.71 d) when treated with a low or high dose of minocycline, respectively. These results indicate that lifespan extension induced by minocycline treatment is not mainly due to germ-free effect, which is consistent with our observation that the same dose of tetracycline treatment as minocycline failed to increase *Drosophila* lifespan (Supplementary Figure 4). It is noted that lifespan extending effects of minocycline were less dramatic in germ-free flies, raising the possibility that minocycline may also influence *Drosophila* lifespan through modulating microbiota.

### Minocycline feeding does not decrease dietary consumption or fecundity

**Figure 4:**
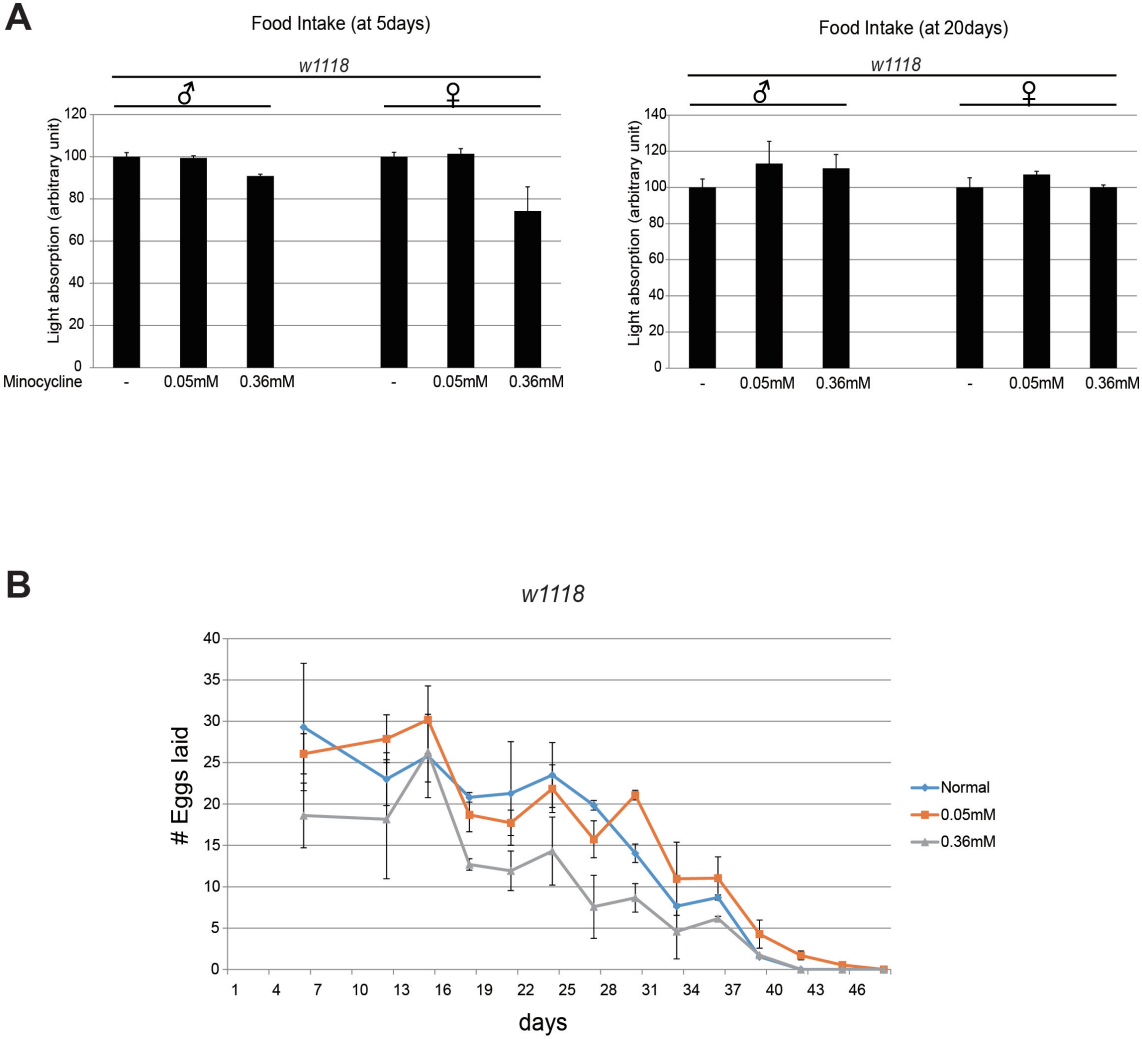
Minocycline treatment barely affects food intake and female fly fecundity **(A)** Food intake did not differ between flies exposed to a low (0.05 mM) or high (0.36 mM) dose of minocycline and flies not exposed to minocycline. Food intake was quantified using blue-dye labeling, the accumulation of which in fly bodies was measured with fly extracts. Two different age groups (5 d and 20 d) were examined. n=10 per replicate. **(B)** Egg numbers did not differ between female flies exposed to a low dose (0.05 mM) of minocycline and flies not exposed to minocycline. The number of eggs laid decreased slightly when flies were exposed to a high dose (0.36 mM) of minocycline. For total number of eggs, 198.8 (normal), 191.4 (0.05mM), 96.4 (0.36mM). n=15 per replicate. Three biological replicates were analyzed. Data are presented as mean ± SEM.

Dietary restriction has been considered a reliable means of extending animal lifespan [18, 19]. It is possible that the minocycline-containing food was less palatable to *Drosophila*, resulting in lifespan extension due to reduced dietary intake. We investigated the food consumption rate using a food dye. We found that the amount of food consumed did not differ significantly overall when the food contained minocycline (Figure 4A), although food intake of 5-day-old flies decreased slightly when the food contained a high dose of minocycline (0.36 mM), a phenomenon not observed in 20-day-old flies. Notably, a low dose of minocycline (0.05 mM), which had exerted the strongest effect on lifespan, did not significantly affect the rate of food consumption in either 5-day-old and 20-day-old flies. This finding negates the possibility that minocycline-induced lifespan extension in *Drosophila* results from reduced food intake.

Some long-lived animals exhibit reduced reproductive activity and hence produce fewer progenies. Lifespan extension following a removal of germline cells has been observed in several animal models [18, 19]. We investigated whether minocycline treatment extends lifespan by attenuating fecundity in flies. Egg counts during the course of aging revealed that fecundity of female flies was slightly decreased by a high dose of minocycline (0.36 mM). However, it was largely unaffected by a low dose of minocycline (0.05 mM), the concentration that exerted the strongest longevity effect (Figure 4B). This finding indicates that the longevity effect of minocycline is not attributable to the inhibition of reproductive activity in *Drosophila*.

### Minocycline feeding increases resistance to oxidative stress

**Figure 5:**
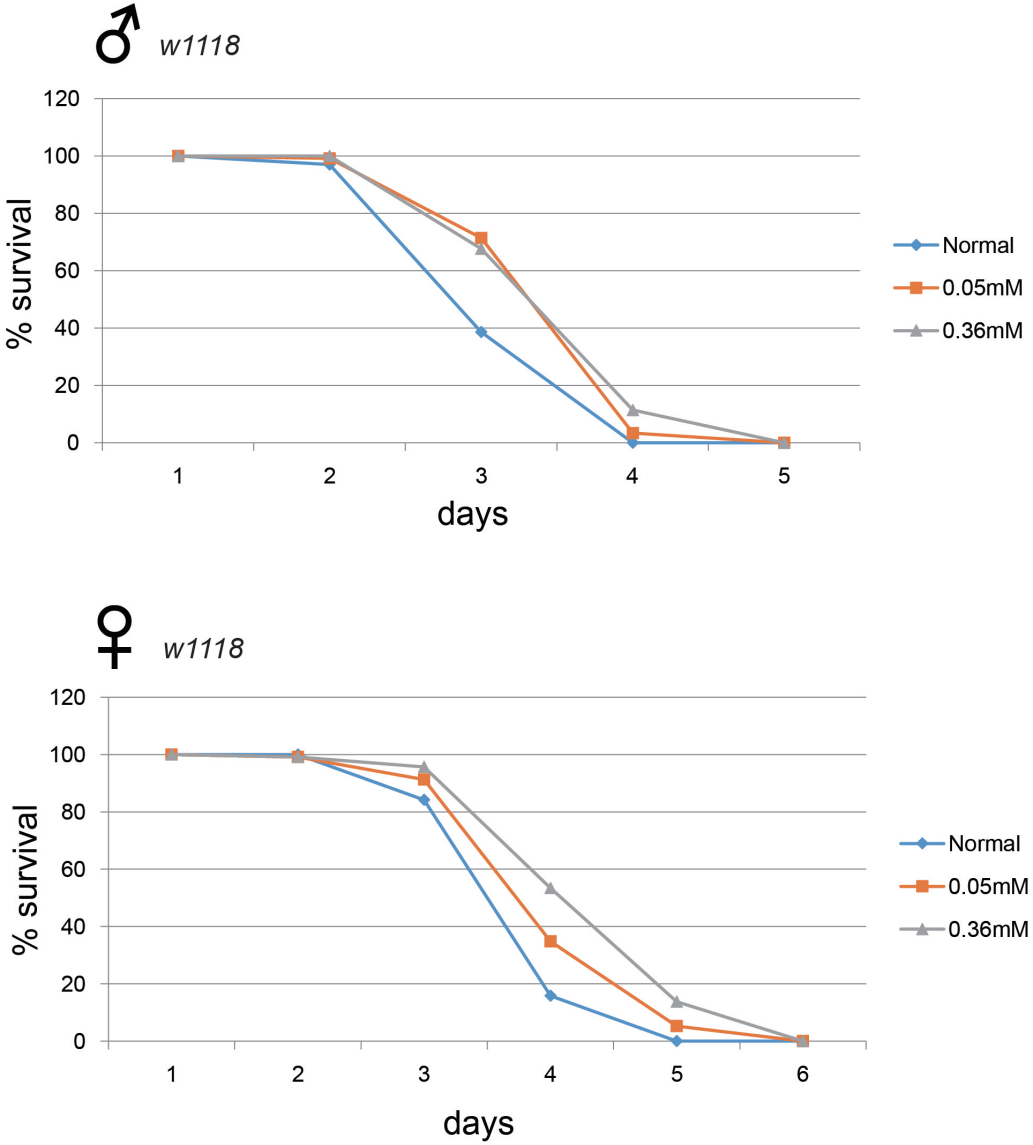
Minocycline treatment increases resistance to oxidative stress Flies fed minocycline showed a greater resistance to hydrogen peroxide than control flies. This phenomenon was observed in both male (*upper panel*) and female (*lower panel*) flies. A high dose (0.36 mM) of minocycline appears to confer greater resistance to oxidative stress. For male flies, n = 101 (normal), n = 119 (0.05 mM), n = 105 (0.36 mM); *p* = 3.7 × 10^−7^ (normal vs. 0.05 mM), *p* = 5.9 × 10^−7^ (normal vs. 0.36 mM). For female flies, n = 101 (normal), n = 115 (0.05 mM), n = 116 (0.36 mM); *p* = 0.0004 (normal vs. 0.05 mM), *p* < 1.0 × 10^−10^ (normal vs. 0.36 mM). *p*-values for lifespan curves were calculated by the log-rank test. All lifespan experiments were repeated with similar results; representative experiments are shown.

Long-lived mutant animals frequently exhibit higher resistance to oxidative stress [18, 19]. We examined whether minocycline treatment could confer resistance to oxidative stress in *Drosophila*. We found that flies treated with minocycline became more resistant to hydrogen peroxide, dying less often than flies not treated with minocycline (Figure 5). This minocycline-induced resistance to reactive oxygen species (ROS) was seen in both male and female flies. Interestingly, fly resistance to hydrogen peroxide appeared to be more robust following a high dose of minocycline (0.36 mM), contrasting with the stronger effect of a low dose (0.05 mM) on longevity.

### Minocycline-induced lifespan extension and ROS resistance are abrogated by *FOXO* mutation

**Figure 6:**
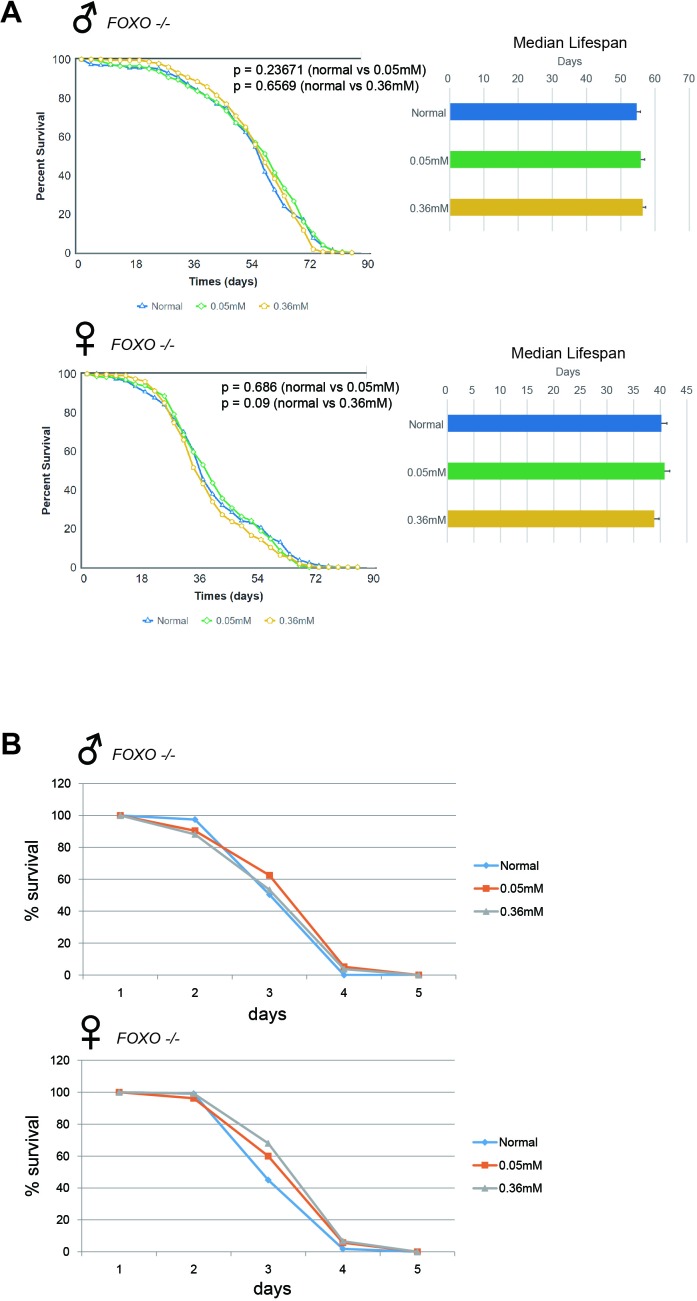
Minocycline fails to extend lifespan and barely increases resistance to oxidative stress in FOXO-null flies **(A)** Minocycline treatment (0.05 mM and 0.36 mM) failed to extend lifespan in *FOXO*-null mutant flies, as shown by the survival curve (*left*) and by quantification of median lifespan (*right*). The failure to extend lifespan was seen in both male (*upper panel*) and female (*lower panel*) flies. For male flies, n = 210 (normal), n = 220 (0.05 mM), n = 223 (0.36 mM); *p* = 0.23671 (normal vs. 0.05 mM), *p* = 0.6569 (normal vs. 0.36 mM). For female flies, n = 223 (normal), n = 228 (0.05 mM), n = 210 (0.36 mM); *p* = 0.686 (normal vs. 0.05 mM), *p* = 0.09 (normal vs. 0.36 mM). *p*-values for lifespan curves were calculated by the log-rank test. All lifespan experiments were repeated with similar results; representative experiments are shown. Data for median lifespan are presented as mean ± SEM. **(B)** Male *FOXO*-null mutants fed minocycline did not differ from controls (no minocycline) in sensitivity to hydrogen peroxide (*upper panel*). Resistance to hydrogen peroxide was slightly higher in minocycline-exposed female *FOXO*-null mutants (*lower panel*). For male flies, n = 119 (normal), n = 136 (0.05 mM), n = 135 (0.36 mM); *p* = 0.0471 (normal vs. 0.05 mM), *p* = 0.7333 (normal vs. 0.36 mM). For female flies, n = 111 (normal), n = 105 (0.05 mM), n = 122 (0.36 mM); *p* = 0.0275 (normal vs. 0.05 mM), *p* = 0.0002 (normal vs. 0.36 mM). *p*-values for lifespan curves were calculated by the log-rank test. All lifespan experiments were repeated with similar results; representative experiments are shown.

The FOXO transcription factor is involved in a variety of cellular processes to regulate organismal physiology, and is a major determinant of animal lifespan [25]. FOXO activation mediates the lifespan-extending effects of attenuated insulin/insulin-like growth factor-like signaling, and elicits cellular and organismal resistance to various stressors, including oxidative stimuli [19, 25]. We wanted to know whether lifespan extension and resistance to oxidative stress induced by minocycline treatment in *Drosophila* involve FOXO. To confirm whether minocycline treatment exerts its lifespan-extending and stress-resistance effects via FOXO, a *FOXO*-null mutant fly (*Foxo^21rev6A^*) was used. We found that minocycline failed to extend lifespan in the *FOXO*-null fly (Figure 6A), in contrast with the observations in *Oregon R* [22, 24], *Canton S* (Figure 1), and *w^1118^* (Figure 2) flies. Failure to extend lifespan was seen in both male and female mutant flies, regardless of *white* gene mutation (Supplementary Figure 5). Minocycline effects on resistance to oxidative stress were also examined in the *FOXO*-null mutant flies. Consistent with the lifespan findings, minocycline-induced resistance to hydrogen peroxide was largely abrogated in *FOXO*-null mutant flies, although a marginal increase in resistance was seen in female flies (Figure 6B). Taken together, these results indicate that minocycline's effects on lifespan and oxidative stress resistance are mediated by FOXO in *Drosophila*.

### Minocycline feeding increases FOXO expression

**Figure 7:**
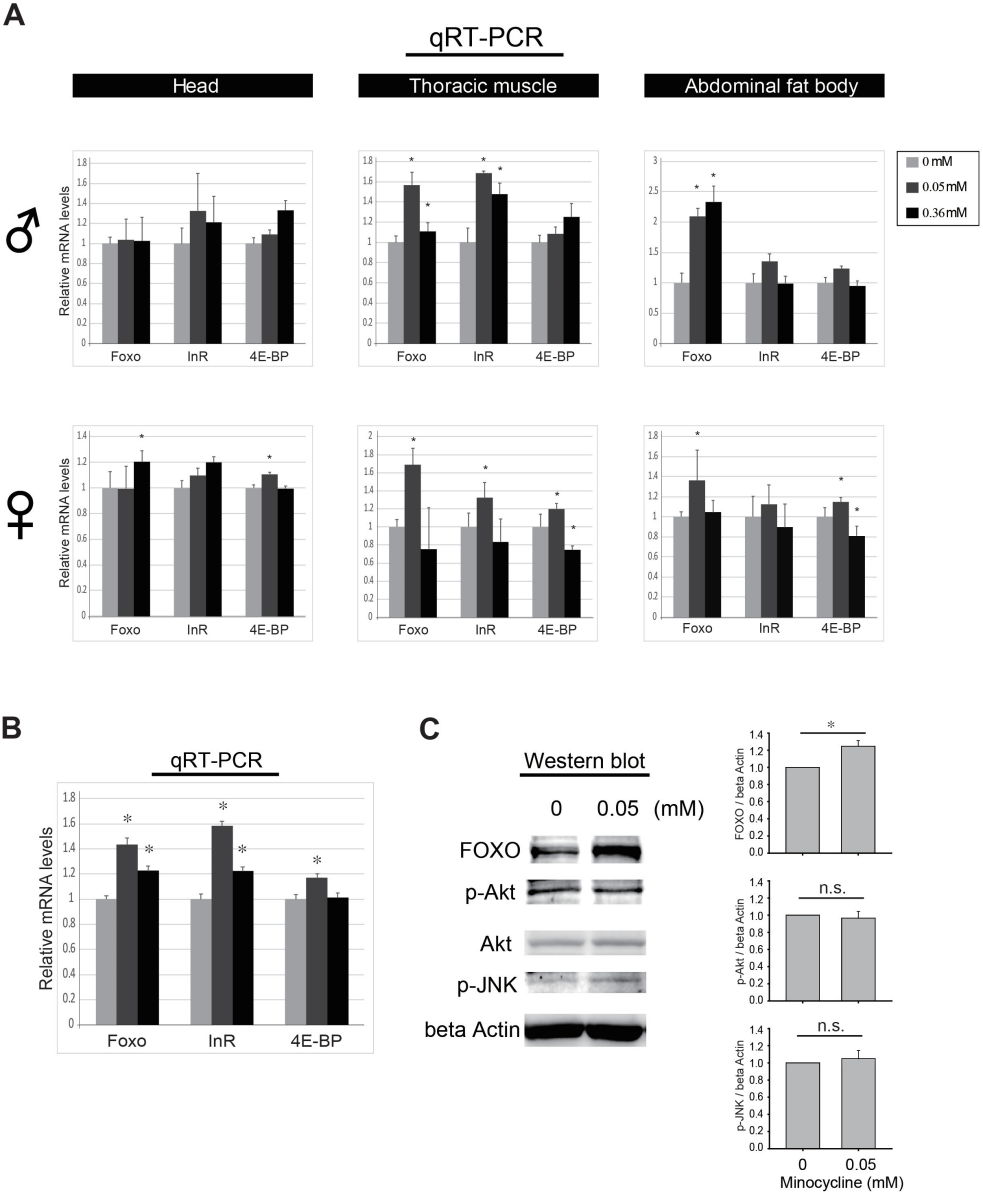
Minocycline treatment increase FOXO activity **(A)** Minocycline treatment for 3 days frequently increases mRNA expression of *FOXO* and its target genes such as *InR* and *4E-BP*. This phenotype is broadly seen across the tissues including head (left panel), thoracic muscle (middle panel), and abdominal fat body tissues (right panel), and is also consistently seen in both male (♂) and female (♀) flies. n=18 (head), n=10 (thoracic muscle), and n=10 (abdominal fat body) per treatment. **(B)** Representation of minocycline's influence on mRNA expression of *FOXO, InR* and *4E-BP* by pooling individual data sets separated by tissues and sexes shown in panel A. **(C)** Minocycline treatment increases the expression of FOXO protein but marginally affects Akt and JNK activity shown by western blot (left panel) and its quantification (right panel). Flies were treated with hydrogen peroxide and indicated concentrations of minocycline for 2 days and abdominal fat bodies were prepared. Similar results were seen in head and thoracic muscle tissues. Three biological replicates were analyzed. Data are presented as mean ± SEM. ^*^p <0.05 when compared to the respective controls (t-test). n.s., not significant.

The critical dependence of FOXO in the minocycline's effects on oxidative stress resistance and lifespan extension led us to test whether the drug treatment has any influence on FOXO activity. We found that there was a general trend in increase of mRNA expression of FOXO target genes, such as *Inr* and *4E-BP*, as well as *FOXO* gene itself when flies were fed minocycline in both normal and stress condition (Figure 7A, 7B, and Supplementary Figure 6). Notably, this trend of FOXO activation was seen across various tissues such as head, thoracic muscle, and abdominal fat body tissues, and this phenomenon was consistently seen in both male and female flies. To further explore the underlying mechanism, the levels of activated form of Akt and JNK in parallel with FOXO protein were examined by western blot. We found that minocycline treatment consistently increased the level of FOXO protein throughout the tissues examined, but the drug treatment marginally affected the level of activated form of Akt and JNK (Figure 7C). Thus, it appears that minocycline increases the activity of FOXO independently of Akt or JNK. Consistent with the effects of minocycline on fly lifespan and FOXO expression, ubiquitous overexpression of FOXO increased the *Drosophila* lifespan (Supplementary Figure 7). Intriguingly, extended lifespan of the fly overexpressing FOXO was further increased by minocycline treatment (Supplementary Figure 7). Since minocycline treatment increases *FOXO* transcript level, this result indicates that minocycline treatment may stimulate FOXO activity in both transcriptional and post-transcriptional manners. Altogether, these results support the notion that minocycline-induced enhancement of FOXO may confer resistance to oxidative stress and extend lifespan in *Drosophila*.

## DISCUSSION

Since minocycline was first produced in the 1960s, it has been used to treat a broad range of symptoms related to bacterial infections such as acne vulgaris. In recent years, attention has been paid to its further effectiveness in treating various pathological conditions not associated with bacterial infections. Many studies have demonstrated that minocycline can suppress inflammatory disorders, tumorigenesis, and angiogenesis [6–9, 26]. Notably, the ability of minocycline to cross the blood-brain barrier encourages the investigation of the drug as a potential therapeutic agent for many neurological disorders, such as Alzheimer's disease, amyotrophic lateral sclerosis, Huntington's disease, and Parkinson's disease [13–17]. Although minocycline's curative potential for various pathological conditions has been shown, the underlying mechanisms are still unclear. In this study, we used *Drosophila* to investigate the effects of minocycline on the aging process and their potential underlying mechanisms. We found that flies treated with minocycline lived longer than controls. These effects were seen in two *Drosophila* strains, and were independent of sex. The longevity effect of minocycline in the *w^1118^* strain was intriguing, since minocycline's effect was previously suggested to be dependent of the kynurenine pathway [22, 23], which is compromised in *w^1118^* strain by mutation of *white*, a gene encoding tryptophan transporter [21].

To explore the underlying mechanism of minocycline's effects, its influences on food intake, reproductive activity, and resistance to oxidative stress were examined as traits often associated with lifespan modification. We found that neither food consumption nor fecundity were significantly affected; however, minocycline increased resistance to an oxidative stimulus, hydrogen peroxide. Notably, minocycline effects on longevity and oxidative stress resistance were largely abolished in *FOXO*-null mutant flies, and the activity of FOXO increased following minocycline treatment. Altogether, these results suggest that minocycline treatment in *Drosophila* enhances FOXO activity, thereby conferring resistance to oxidative stress and extending lifespan.

Several molecular targets of minocycline have been proposed to explain the non-antibiotic properties of the drug. It has been shown that minocycline physically interacts with apoptotic protease activating factor 1 (Apaf-1), which inhibits caspase activation and cellular apoptosis [27]. Increased production of ROS in numerous pathological conditions may aggravate cellular homeostasis and damage the cell [28]. Because of its multi-substituted phenol ring, minocycline can scavenge free radicals, protecting against oxidative stress [29]. However, our results provide an alternative explanation for the oxidative stress resistance induced by minocycline: The drug increases the activity of the FOXO transcription factor in animal cells, thereby rendering the organism more resistant to oxidative stress. In line with this, minocycline has also been shown to inhibit some enzymes, such as metalloproteinases and oxygenases, that generate ROS [30, 31], which might explain the marginal increase in oxidative stress resistance induced by minocycline seen in *FOXO* null mutant (Figure 6B).

Our findings may also provide a plausible explanation for other non-antibiotic effects of minocycline. The drug has been shown to have tumor-suppressive properties in leukemia, prostate cancer, and ovarian cancers [32–34], which may be partly explained by minocycline's effect on activating FOXO, a well-known tumor suppressor. Future studies should characterize the molecular mechanism responsible for minocycline's effect on FOXO activity.

## MATERIALS AND METHODS

### Fly rearing and lifespan measurement

The *Drosophila melanogaster* strains, *w^1118^, Canton S, UAS-FOXO, heat shock Gal4* (*HS-Gal4*) were obtained from Bloomington stock center (University of Indiana, Bloomington). *FOXO^21rev6A^* is the *FOXO* null allele in which 2nd site lethal mutation was removed from *FOXO^21^* (A gift from Dr. Marc Tatar). All flies used in this study were reared on the same diet and same environmental conditions: ambient temperature of 25°C, with 60% humidity and 12-h light and dark cycles, on standard yeast-glucose medium (86.2 g/L glucose, 40.8 g/L cornmeal, 62.4 g/L dried yeast, and 9.3 g/L agar. Tegosept and propionic acid were added as preservatives). Minocycline stock solution (45 mM) was prepared by dissolving minocycline hydrochloride (M9511, Sigma Aldrich) in distilled water and mixed in medium with appropriate final concentrations. Tetracycline stock solution (45 mM) were similarly prepared (T7660, Sigma Aldrich). More than 210 flies per treatment were collected at 3 days after eclosion with the distribution of 15 flies per vial. Dead flies were counted every 3 d, when remaining flies were transferred to fresh food vials, a practice that was continued until all flies were dead. This process constituted one biological replicate, and was repeated at least three times to yield three independent biological replicates. Lifespan and mortality rate analyses were performed using Online Application for the Survival Analysis of lifespan assays 2 (OASIS 2) [35] and the p values were calculated using the log-rank test.

### Establishment of germ-free fly

Germ-free flies were made according to the method described in a previous study [36] with slight modification: Germ-free animals were generated by decontaminating dechorionated embryos for 1min 30sec in 2.7% sodium hypochlorite. Embryos were subsequently washed twice in 70% ethanol, followed by three washes with sterile water. The embryos were then maintained with axenic foods and routinely checked for bacterial contamination by culturing the homogenates on nutrient-agar plates.

### Measurements of food intake

A food intake assay was performed following the method described in a previous study [37] with slight modification: Flies were cultured for 90 minutes in food containing 0.5% Bromophenol Blue. 10 fly bodies were homogenized in distilled water and supernatant was used for measuring the absorbance at 625nm. Three biological replicates were analyzed per treatment.

### Measurements of fecundity

15 female flies were used per batch and male flies were removed after 3 days of mating. Each vial contained one female fly and it was transferred every three days to fresh food vial. The number of eggs laid was counted every three days until no egg was laid. The experiment was repeated to yield three biological replicates per treatment.

### Measurements of locomotor activity

Climbing ability of flies was measured by using rapid iterative negative geotaxis (RING) assay [38] with slight modifications. Adult fruit flies were exposed to normal food or minocycline contaminated food and then transferred to new vials —15 males and 15 females in each vial. These vials were loaded into the equipment and tapped on a table three times in rapid succession to initiate a negative geotaxis response. The positions of flies were captured by a digital camera 5 sec after initiation of behavior, and the number of flies that climbed above the standards (30 mm from the bottom) was counted. After each trial, the flies were allowed 1 min of recovery from shock. These cycles were conducted three times with 10 replicates in each group.

### Measurements of resistance to hydrogen peroxide

1-day-old flies of the indicated genotypes were mated for 3 days, then were segregated by females and males. The flies were transferred to medium containing 3% hydrogen peroxide (H0298, Samchun chemical) and various concentrations (0mM, 0.05mM, or 0.36mM) of minocycline. Dead flies were counted once a day. The experiment was repeated to yield three biological replicates per treatment.

### RNA preparation and quantitative RT-PCR (qRT-PCR)

Adult flies previously anaesthetized by ice were dissected in 70% ethanol. Head and thorax were separated and prepared. Abdominal fat bodies were prepared by removing midgut and gonadal tissues from abdominal carcasses. Prepared samples were quickly transferred to Trizol solution (RNAiso Plus, Takara) and ground for RNA preparation. RNA was extracted following the manufacturer's recommended instructions. cDNAs were synthesized using RevertAid Reverse Transcriptase (Thermo Scientific). PCR was performed using the CFX Connect Real-Time PCR Detection System (Bio-Rad) and SYBR Premix Ex Taq (TaKaRa). All interested mRNA levels were calculated as a relative fold-change over *Rp49* mRNA. The comparative cycle threshold (Ct) method was applied to estimate mRNA levels. The following PCR primers were used: *InR*, 5′-GGA TGC GTG ATC GAT AAG AA-3′ and 5′-CTG ATA ATA GCC TTT CGG ACA G-3′; *4E-BP*, 5′-ATG CAG CAA CTG CCA AAT C-3′ and 5′-CCG AGA GAA CAA ACA AGG TGG-3′; *FOXO*, 5′-CAG AAT GCG AAT GCA GCC AA -3′ and 5′-CCA ATG GCA TGC GTG ATG AG-3′; *Rp49*, 5′-AGG GTA TCG ACA ACA GAG TG-3′ and 5′-CAC CAG GAA CTT CTT GAA TC-3′.

### Western blot

Head, thorax and abdomen in adult fly were homogenized in RIPA lysis buffer (Thermo Fisher Scientific, Cat. # 89900) containing the protease and phosphatase inhibitor cocktail (Thermo Fisher Scientific, Cat. # 78449). The isolated protein samples pooled from 25 flies, were quantified by the Bradford assay and separated in 10% poly-acrylamide gel. The transferred Immobilon^TM^ PVDF Membrane (Millipore) was blocked for 1 h at room temperature (RT) in TBST containing 5% BSA and incubated overnight at 4°C. The anti-phospho-AKT (Cell signaling, Cat. #4054), anti-pan-AKT (Cell signaling, Cat. #9272), anti-phospho-JNK (Cell signaling, Cat. #9255) anti-dFOXO (Cosmo Bio, Cat. THU-A-DFOXO), and anti-Actin (Cell signaling, Cat. #4967) was diluted to 1:1000 with 5% BSA in TBST when used as a primary antibody. After washing, the membrane was incubated with 5% skim milk in TBST containing HRP-conjugated secondary antibody (1:10000) at RT for 1 h. The HRP signal was generated following the ECL substrate treatment and analyzed by the ChemiDoc mini-HD6 system (UVITEC).

## SUPPLEMENTARY FIGURES


