# Supplementary material for: Minocycline treatment increases resistance to oxidative stress and extends lifespan in Drosophila via FOXO
Source: Oncotarget. 2017 Sep 23;8(50):87878–90. doi: 10.18632/oncotarget.21224 (PMC5675679; doi:10.18632/oncotarget.21224)
Supplement: Supplementary file 1 [file oncotarget-08-87878-s001.pdf]

Minocycline treatment increases resistance to oxidative stress and extends lifespan in *Drosophila* via FOXO

SUPPLEMENTARY MATERIALS

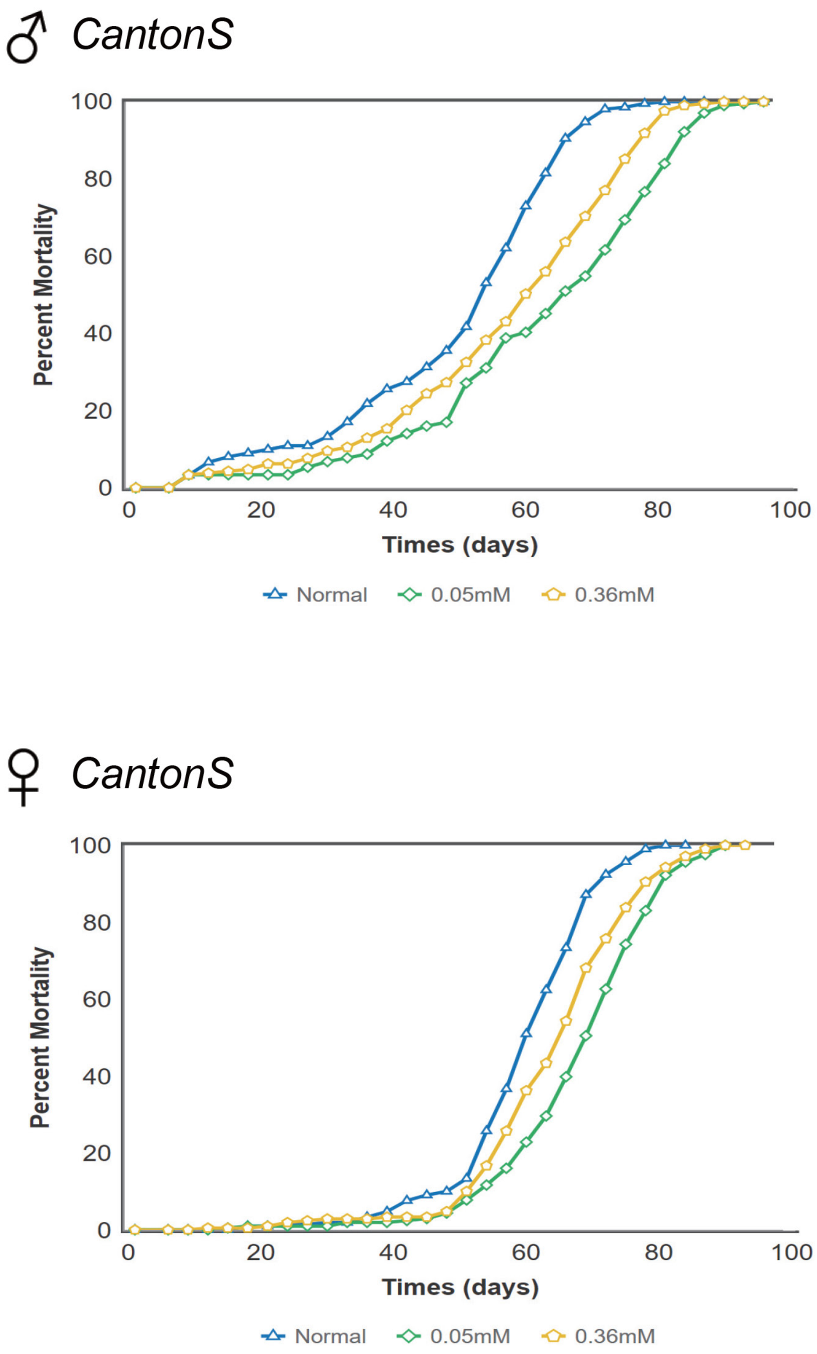

Supplementary Figure 1: Representation of lifespan data in Figure 1 by plotting mortality rate. Mortality rates increase as flies age, which are decreased by minocycline treatment.

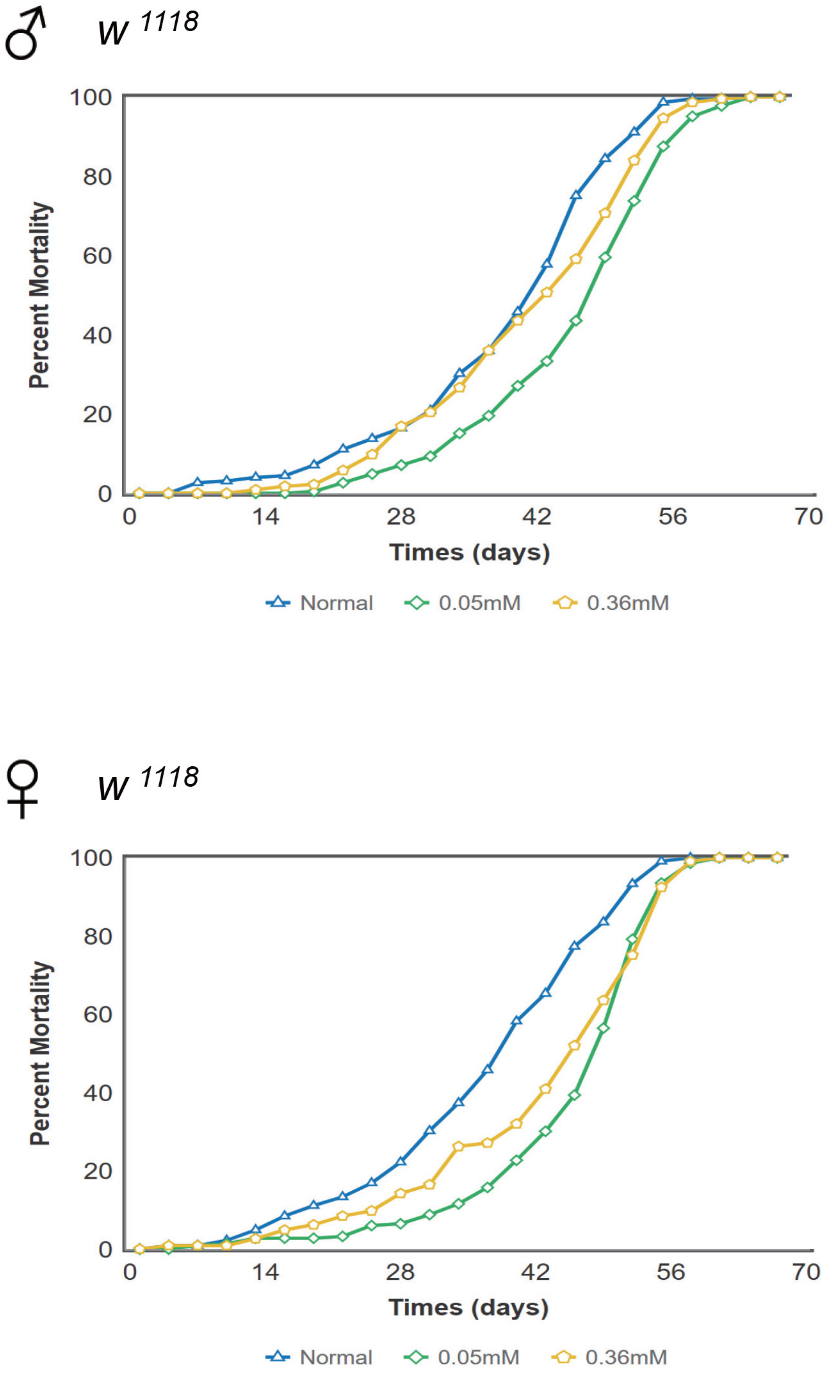

Supplementary Figure 2: Representation of lifespan data in Figure 2 by plotting mortality rate. Mortality rates increase as flies age, which are decreased by minocycline treatment.

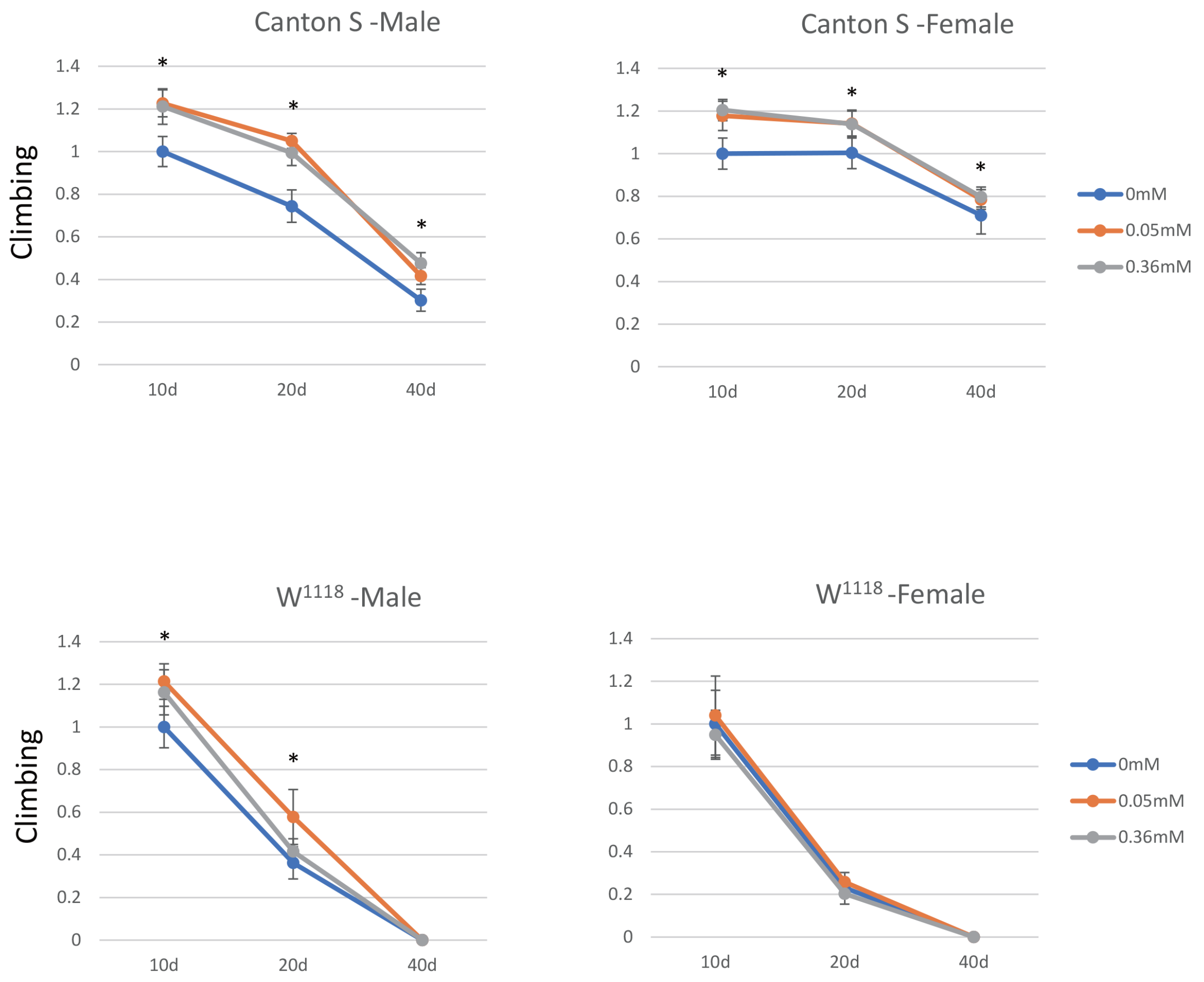

Supplementary Figure 3: Minocycline treatment generally increases locomotor activity in flies. Flies that climbed up to specific height of the vial were counted at 3 points of days as flies aged. Stimulation of locomotive activity is more pronounced in male flies than in female flies and in Canton S flies than in w1118 flies. Minocycline treatment failed to increase the activity of w1118 female flies. Data are presented as mean ± SEM. *p <0.05 when compared to the respective controls (t-test).

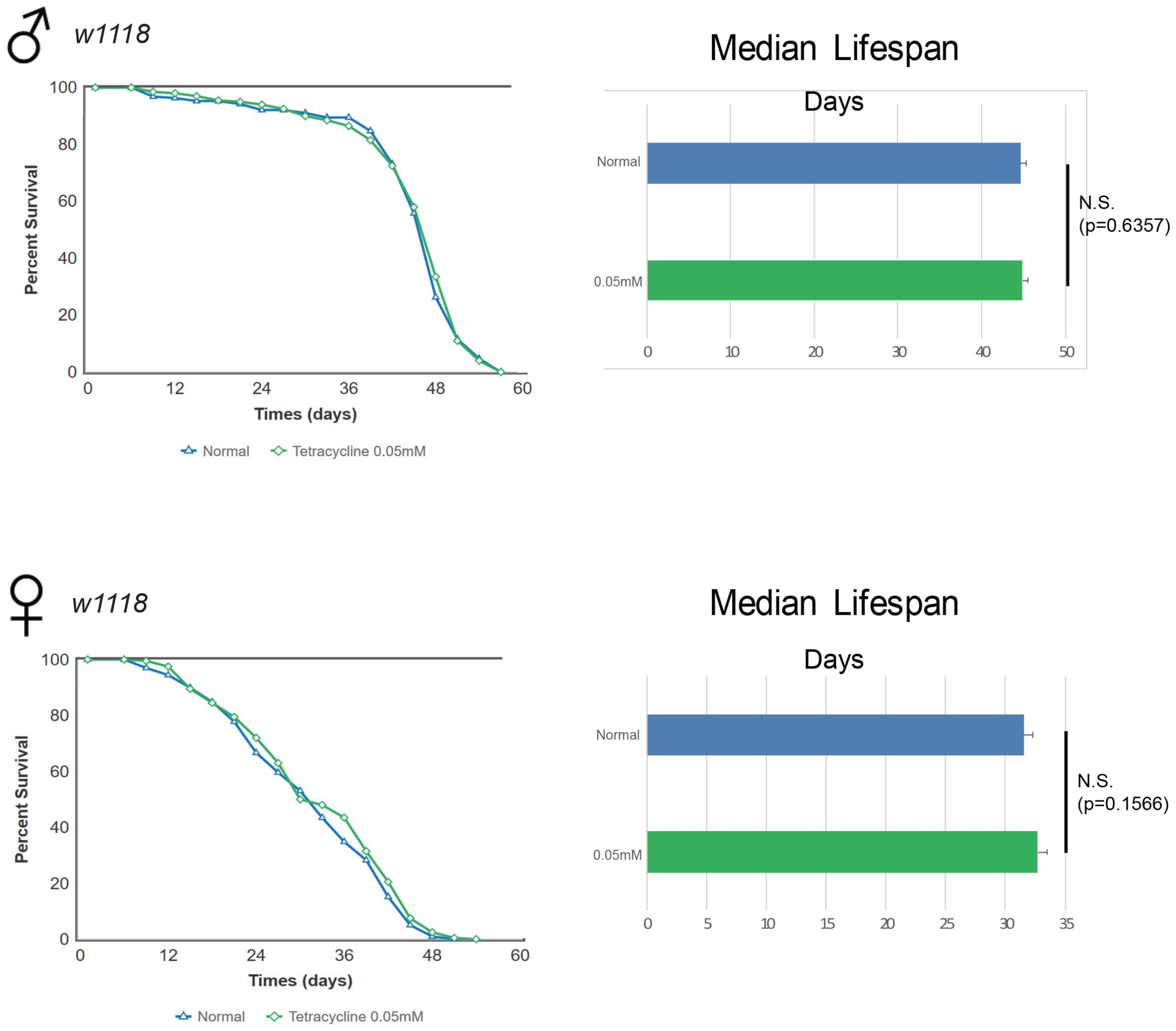

Supplementary Figure 4: Tetracycline treatment marginally affects fly lifespan. Tetracycline treatment at 0.05mM failed to increase the lifespan of male or female w1118 flies. For male flies, n=220 (normal), n=232 (0.05mM). For female flies, n=180 (normal), n=192 (0.05mM). All lifespan experiments were repeated with similar results; representative experiments are shown. Data for median lifespan are presented as mean ± SEM. N.S., not significant.

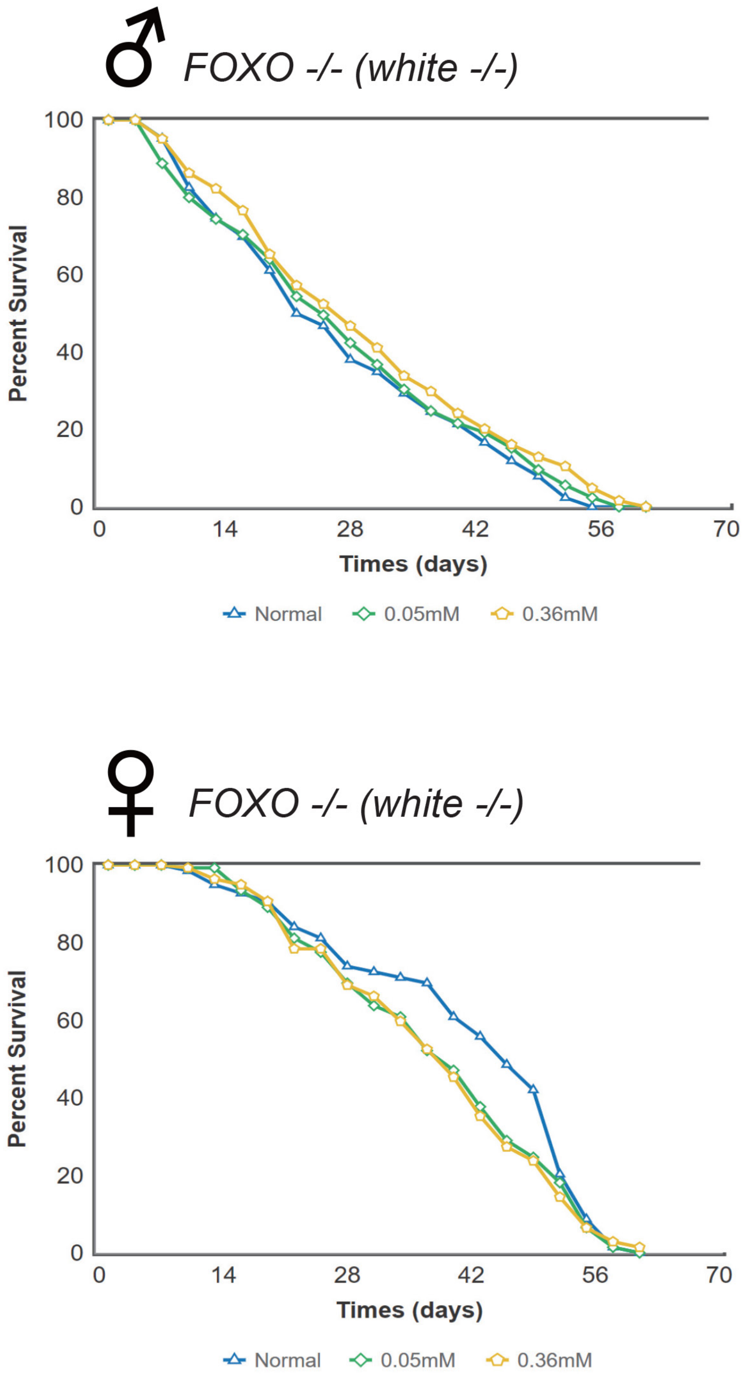

Supplementary Figure 5: Minocycline fails to extend lifespan of FOXO-null flies having white gene mutation. Minocycline at two different doses (0.05 mM and 0.36mM) failed to extend lifespan in FOXO-null mutant flies having white gene mutation. The failure to extend lifespan was seen in both male (upper panel) and female (lower panel) flies. For male flies, n = 150 (normal), n = 163 (0.05 mM), n = 153 (0.36 mM). For female flies, n = 170 (normal), n = 165 (0.05 mM), n = 161 (0.36 mM).

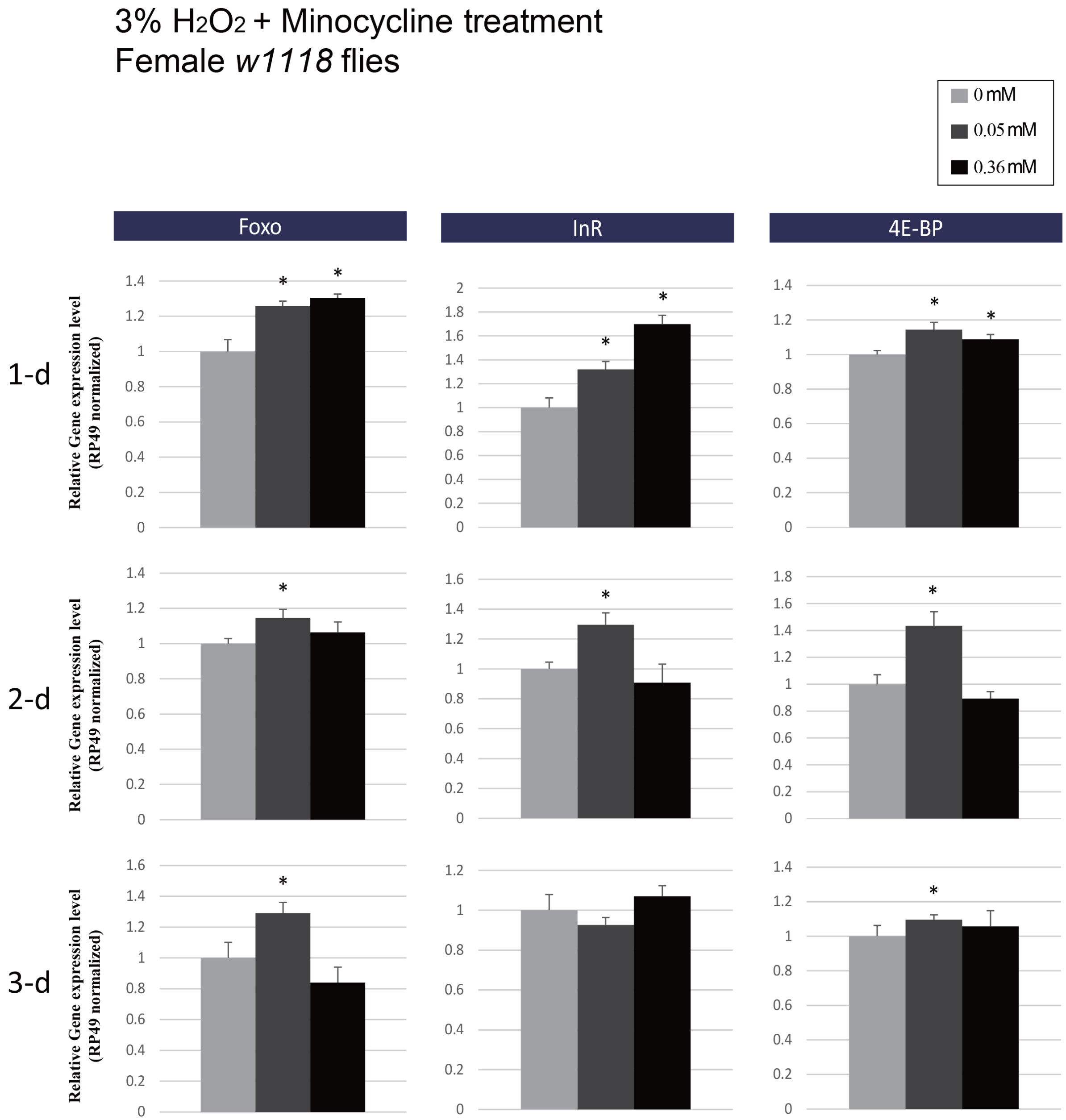

Supplementary Figure 6: Kinetics of the mRNA expression of FOXO and its target genes in response to minocycline treatment. Flies were treated with the indicated doses of minocycline and total RNA was prepared during 3 days in ROS stress condition. mRNA levels of FOXO, InR, and 4E-BP genes were measured using qRT-PCR. Data are presented as mean ± SEM. *p <0.05 when compared to the respective controls (t-test).

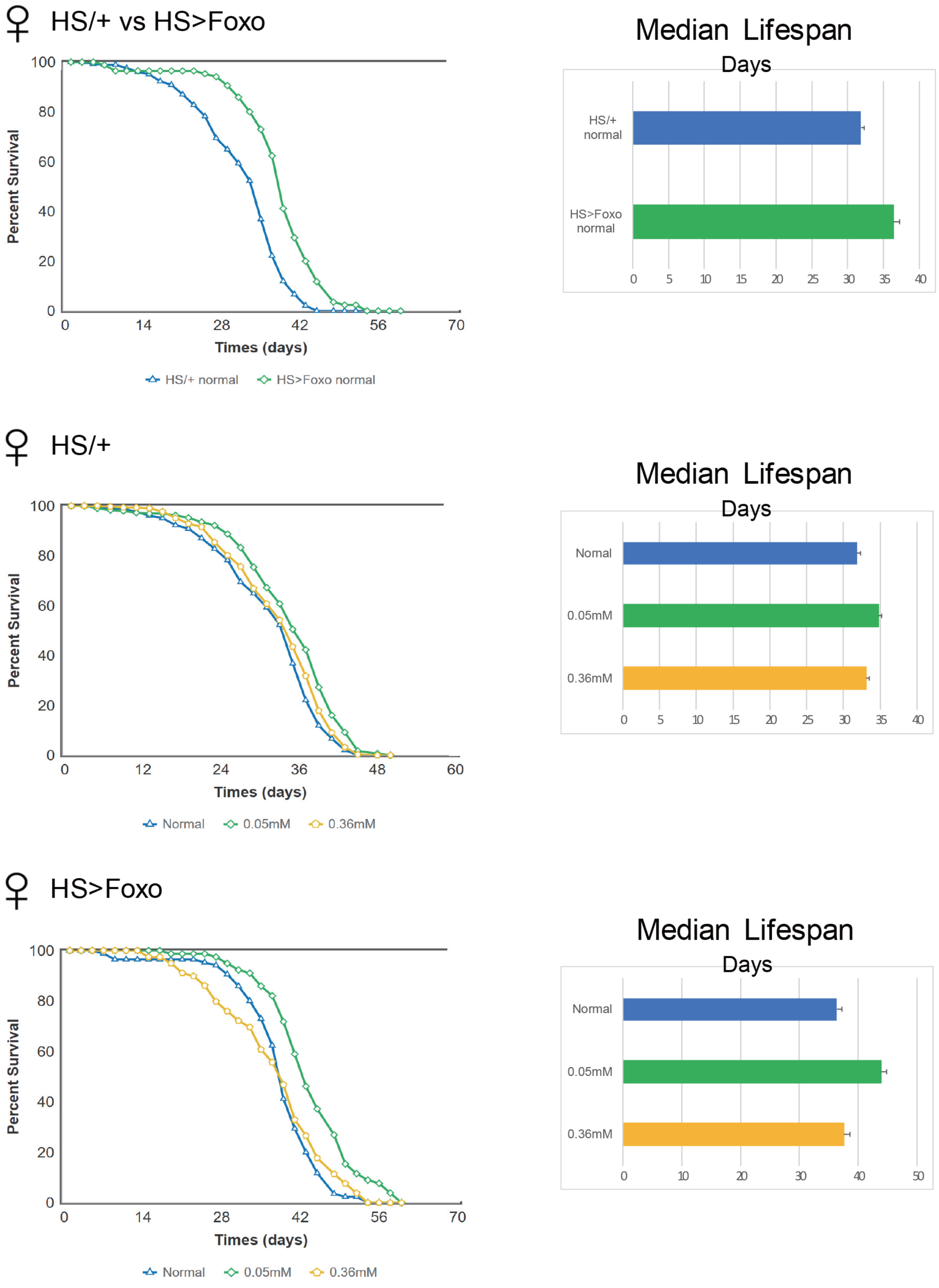

Supplementary Figure 7: Ubiquitous overexpression of FOXO increases fly lifespan, which is further extended by minocycline treatment. Ubiquitous overexpression of FOXO by heat shock Gal4 (HS) induced expression of FOXO with incubation at 29°C increases fly lifespan (Upper panel). Control flies having only gal4 transgene shows increased lifespan upon minocycline treatment (Middle panel). The flies ubiquitously overexpressing FOXO shows increased lifespan upon minocycline treatment (Lower panel). More than 130 flies in each of the test groups were observed. All lifespan experiments were repeated with similar results; representative experiments are shown. Data for median lifespan are presented as mean ± SEM.
